# Interstitial Fluid in Gynecologic Tumors and Its Possible Application in the Clinical Practice

**DOI:** 10.3390/ijms19124018

**Published:** 2018-12-12

**Authors:** Blendi Ura, Giovanni Di Lorenzo, Federico Romano, Lorenzo Monasta, Giuseppe Mirenda, Federica Scrimin, Giuseppe Ricci

**Affiliations:** 1 Institute for Maternal and Child Health—IRCCS “Burlo Garofolo”, 34137 Trieste, Italy; giovanni.dilorenzo@burlo.trieste.it (G.D.L.); federico.romano@burlo.trieste.it (F.R.); lorenzo.monasta@burlo.trieste.it (L.M.); giuseppe.mirenda@burlo.trieste.it (G.M.); federica.scrimin@burlo.trieste.it (F.S.); giuseppe.ricci@burlo.trieste.it (G.R.); 2 Department of Medical, Surgery and Health Sciences, University of Trieste, 34137 Trieste, Italy

**Keywords:** tumor interstitial fluid, gynecologic tumors, proteomics, biomarker

## Abstract

Gynecologic cancers are an important cause of worldwide mortality. The interstitium consists of solid and fluid phases, situated between the blood vessels and cells. The interstitial fluid (IF), or fluid phase, is an extracellular fluid bathing and surrounding the tissue cells. The TIF (tumor interstitial fluid) is a dynamic fluid rich in lipids, proteins and enzyme-derived substances. The molecules found in the IF may be associated with pathological changes in tissues leading to cancer growth and metastatization. Proteomic techniques have allowed an extensive study of the composition of the TIF as a source of biomarkers for gynecologic cancers. In our review, we analyze the composition of the TIF, its formation process, the sampling methods, the consequences of its accumulation and the proteomic analyses performed, that make TIF valuable for monitoring different types of cancers.

## 1. Introduction

Gynecological cancers are an important cause of worldwide mortality. Based on the WHO (World Health Organization) records, the annual incidence of ovarian, uterine and cervical tumors is approximately one million people, accounting for up to 500,000 cancer deaths every year [1]. Reaching a diagnosis of gynecological cancer is still a big challenge. Diagnostic procedures nowadays are mainly based on instrumental imaging techniques, which usually result in late diagnosis, when the disease has already reached an advanced stage. Recently, researchers have investigated molecular-based diagnostic techniques, such as the use of nanoparticles [2] or the isolation of biomolecular pathology markers that can eventually lead to an early diagnosis [3]. One hypothesis that is currently being investigated by the scientific community is the analysis of the tumor interstitial fluid (TIF) for the identification of biomarkers, and the consequent improvement of diagnostic procedures, prognosis and choice of treatment [4]. The interstital fluid (IF) is the fluid phase of the interstitium, surrounding the cells. It is generated by trans-capillary filtration and cleared mainly by lymphatic vessels [5,6,7]. The slow flow of the IF from capillaries to lymph vessels provides transport of nutrients to the cells in the tissue and, at the same time, transports waste products from the cells to the lymph vessels. Due to the balance between formation and clearance of the IF, there is no net accumulation of fluid, and the pressure is kept constantly low (about 0 mmHg) [8,9,10].

In solid tumors, due to leaky vessels and impaired lymph drainage, fluid accumulates and causes increased fluid pressure. In the central part of the tumor, the IF flow diminishes, leading to reduced waste removal [11,12]. The angiogenic growth of a new vascular system or vessels, net in an expanding tumoral tissue that is stimulated by different substances secreted by neoplastic cells, such as the vascular endothelial growth factor (VEGF) [13,14]. Also, the intravasal compartment of the new vascular tumoral system is enriched by cytokines and chemotactic factors that attract tumoral cells and stimulate their growth [15]. The studies published on TIF analysis in gynecologic cancers are very heterogeneous, as they investigate different aspects and factors. Regarding uterine cervical cancers, what has been mainly studied is the role of TIF pressure and how it can influence the patient’s therapy response, in the context of the overall survival (OS) and the disease-free survival (DFS). On the other hand, researches who have published on ovarian and endometrial cancers have mainly evaluated the isolation of early oncological biomarkers and how to use them as hypothetical early screening tests in clinical practice.

The present study aims to accurately review the role of the TIF in relation to neoplastic pathologies, more specifically to gynecological cancers, and to investigate how its role can be exploited in the clinical practice.

## 2. The Tumor Microenvironment

**Figure 1:**
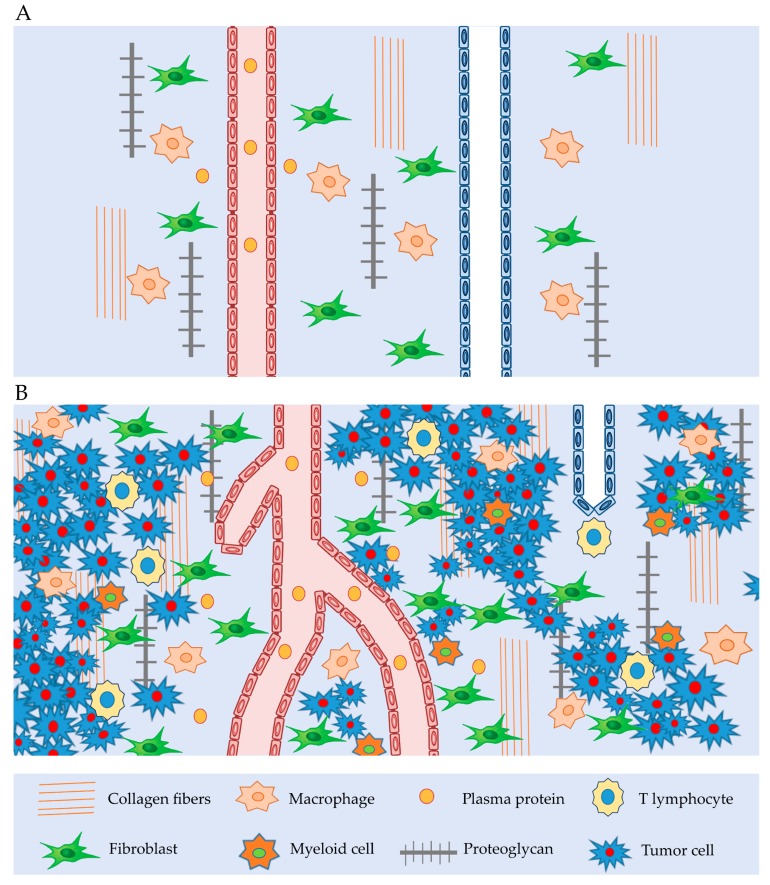
Interstitium tissue: (**A**) Normal interstitium tissue with lymphatic and blood vessel, macrophages, fibroblast, collagen fibers, proteoglygan. (**B**) Tumor interstitium tissue with reduced lymphatic vessel, winding blood vessel, macrophages, fibroblasts, increased amount of extravasated plasma proteins, T-lymphocyte, myeloid cells, collagen fibers, proteoglygan and tumor cells, all of which induce to an increase in tumor interstitial pressure.

The interstitial compartment is comprised of connective and supporting tissues, located outside blood and lymphatic vessels, and parenchymal cells. The main components of the extracellular matrix (ECM) are glycosaminoglycans (GAGs), collagen fibers, plasma proteins and salt. In cancer, the ECM is generally deregulated, facilitating cellular transformation and the production of metastases. An abnormal ECM influences the normal activity of stromal cells, promoting inflammation and angiogenesis [16]. Generally, the components of the tumor interstitium (Figure 1) are the same as those of the normal interstitium, but with some differences. The main difference is that the tumor stroma is “dynamic” in supporting tumor growth [17]. Indeed, a large number of tumor-associated macrophages (TAM) in the tumor interstitium induces the production of inflammatory and pro-angiogenetic factors, such as epidermal growth factor (EGF); vascular interleukin-8 (IL-8) and VEGF-A, also matrix metalloproteinases (MMPs), cathepsins, serine proteases, leading to tumor metastatization [7]. Curiously, the infiltration of cytotoxic T lymphocytes is associated with the production of interferon γ (IFγ) and interleukin-2 (IL-2), which is associated with inhibition of tumor growth [18].

In addition to TAM and T lymphocytes that have a key role in tumor progression, the myeloid cell population can suppress T-lymphocyte activity by the expression of arginase 1 (ARG1) and nitric oxide synthase 2 (NOS2) [9]. Endothelial cells are very important for tumor development and progression. They stimulate angiogenesis to provide nutritional support for the growth of the tumor. They are also fundamental in controlling leukocyte recruitment, tumor cell behavior and metastasis formation [19]. Thus, the tumor cell produces a high amount of growth factors and cytokines [16]. Cancer cells express a high amount of VEGF, leading to high-microvascular permeability and extravasation of plasma proteins such as fibrin, attracting endothelial cells, inflammatory cells and fibroblasts [4]. The TAMs, stroma cells, and fibroblasts are important for the secretion of angiogenetic factors, and contribute to tumor angiogenesis, influencing the tumor stroma structure and function [20,21,22].

## 3. Tumor Interstitial Fluid

The abnormal tumor interstitium represents a great challenge in the study and treatment of cancer [23]. In the tumor microenvironment, cancer cells produce angiogenic factors [24], cytokines [25] and growth factors [26]. In recent years, a great deal of attention has been put in the study of the TIF to understand the pathophysiology of tumors and to identify new therapeutic approaches [27].

The IF composition seems to influence both cell growth and the invasive potential of tumoral cells [28]. TIF flow activates some superficial chemokine cell receptors such as chemokine receptor type 4 (CXCR4) [4] and migration factors. Cancer growth not only depends on the intrinsic invasive potential of tumor cells, but also on the characteristics of its microenvironment. It influences the communication between cells themselves, between tumoral cells and both healthy surrounding tissues and the systemic environment [29]. Therefore, the composition of TIF presents an important source of oncological markers and its molecular analysis will likely increase our knowledge about tumoral pathogenesis. New therapeutic and diagnostic approaches have been recently studied in regards to tumoral pathologies by targeting and modifying TIF composition, with the aim of improving anti-neoplastic therapies [30,31]. For example, the high oncotic pressure in the TIF negatively influences the diffusion of anti-neoplastic drugs into the tumoral mass, and VEGF seems to interfere with this mechanism [4]. The TIF is expected to be enriched with proteins secreted by the endoplasmic reticulum (ER), tumor cell-derived exosomes [32,33] spread by membrane vesicles, or externalized by plasma membrane transporters [28]. Exosomes are 20–200 nm round membrane vesicles, which contain specific proteins and RNA, and induce significant cellular behavioral changes in the receiving cell [34]. Exosomes are released by cells in proximal body fluids, and have been described in urine [35], blood [36], saliva and other body fluids. Exosome signaling is essential in almost all steps necessary for the progress of carcinomas, from primary tumors to metastases [37].

## 4. Biophysical Properties of TIF

Starling et al. [38] for the first time described the biophysical properties of the TIF based on the principle of fluid exchange. Their experiment suggested that the capillaries are semipermeable membranes, and that their filtration is governed by the imbalance between hydrostatic forces and colloid osmotic pressure (COP). Levick et al. [39] made further progress, by generating an equation for fluid filtration applicable to tumors:

J_V_/A = L_P_[(P_C_ − P_if_) − ð (π_p_ − π_i_)]

where J_V_ is the volume filtration rate, A is the endothelial area, L_P_ is the hydraulic conductivity of the capillaries, P_C_ is the capillary hydrostatic pressure, P_if_ is the interstitial fluid pressure, ð is the capillary reflection coefficient, π_p_ is the plasma protein colloid osmotic pressure, and π_i_ is the interstitial colloid osmotic pressure. It is known that P_if_ is higher in solid malignant tumors than in normal tissues [40]. In tumor tissues, measured P_if_ ranges from 5 to 50 mmHg, while in normal tissues it ranges from −3 to +3 mmHg [41,42]. Milosevic et al. [43] measured P_if_ by probe measurements in tumors of cervical cancer patients that underwent radiotherapy or radiotherapy plus concurrent cisplatin. They found that only high P_if_ patients benefited from the adding of a chemotherapeutic drug like cisplatin in combination with radiotherapy. In another study, Milosevic et al. [44] measured tumor P_if_ by a customized needle probe in 102 patients with cervix cancer treated with radiotherapy alone. They found that patients with high P_if_ were significantly more likely than those with low P_if_ to have a recurrence after radiotherapy and to die of progressive disease, independently of clinical prognostic factors.

In patients with cervical cancer following radiation therapy, Seung-Gu Y et al. [45] investigated tumor interstitial fluid pressure as a prognostic factor for recurrence-free survival. For this purpose, P_if_ was measured in 55 cervical cancer patients who received radiation therapy. The P_if_ measurements were made before radiation therapy (pre-radiation therapy) and after radiation therapy (mid-radiation therapy) using a modified wick-in-needle technique. They found that the mid-radiation therapy P_if_ measurement may be useful in predicting radiation therapy responses in cervical cancer.

Hompland et al. [46] evaluated different survival prediction factors in 62 cervical cancer patients. The authors studied the role of increased TIF and the flow velocity of the peritumoral fluid (Vo) which should be directly associated with TIF pressure in the tumoral mass. First of all, Vo had higher values in the lymph nodes of metastatic patients. Conversely, Vo did not appear to be linked with tumor stage, size and histology, or with patients’ age. According to the authors, Vo is an independent prognostic factor for overall survival (OS) and disease-free survival (DFS), depending on: The mass position, tumoral tissue density, efficiency of the lymphatic drainage system, and the hydraulic conductivity of the peritumoral tissue. It is still not clear how P_if_ and Vo could influence the prognosis of cervical cancer patients.

However, there are tumors like pancreatic cancer, in which P_if_ can be very high (99 mmHg), indicating a high degree of malignancy [47].

As mentioned, high TIF in tumors is related to high permeability [48], as a consequence of the lack of lymphatic drainage in the tumor core [49], and the effect of growth factors such as VEGF and transforming growth factor-B (TGF-B) [41]. Eigenmann et al. [50] showed that even large proteins could penetrate the tissue and reach the interstitium. For the first time, they used a physiologically-based pharmacokinetics (PBPK) model to measure tissue interstitial pharmacokinetics (PK) for monoclonal antibodies. They found that antibody interstitial concentrations are tissue-specific, dependent on capillary structure, and reach relatively high interstitial concentrations. To date, few experiments have measured the P_if_ in human cancers, and the algorithms developed are not yet able to accurately measure it. Further efforts are needed to better recognize the factors leading to the increase in the P_if_, because this microenvironment is one of the major obstacles for anti-cancer therapies [51,52].

## 5. TIF Isolation Methods

As mentioned above, the complexity of the interstitium and the large number of molecules involved poses the problem of having to identify methods that reflect the fluid microenvironment of the tissue cells. IF is not readily available and several methods have been developed for its isolation [53,54]. The methods can be divided according to how the TIF is isolated in vivo: Capsule implantation, wick-in-needle technique, microdialysis, glass capillary, and capillary ultrafiltration. Besides these, there are other techniques to obtain TIF from fresh tissue specimens: Tissue centrifugation and tissue elution. Below we describe the most frequently used techniques for the isolation of TIF.

### 5.1. Capsule Implantation

The capsule method was developed by Guyton et al. [55] for measuring interstitial fluid pressure, allowing the possibility of obtaining a specific amount of liquid for analysis. Initially, a perforated capsule is introduced resulting in the extravasation of blood, followed by an inflammatory reaction over the next 2–3 h. After this period, inflammation is reduced and the pressure can be measured with a needle introduced into one of the perforations. Gullino et al. [56] created a capsule to measure the pressure and to obtain an amount of TIF. Their intent was to create a tumor model behaving like an organ connected to the host by a vein and an artery. They thus planted tumor cells on a rat ovary to obtain a tumor mass which could be studied as an isolated organ. With the growth of tumor cells over normal ovarian cells, a capsule was inserted to obtain the interstitial fluid and pressure. This method is useful for obtaining TIF throughout tumoral evolution, verifying its composition, its pressure and the changing of biochemical parameters, such as glucose and pH.

### 5.2. Microdialysis

Microdialysis is a method used extensively in pharmacology [57,58] and TIF proteomic studies. The method is based on the passive diffusion of substances across a semipermeable membrane. The membrane has a molecular range of 20–100 kDa [59]. Membranes with cut-off of 20 kDa are used for small molecules like metabolites or small peptides, while membranes with cut-off of 100 kDa are used for macromolecules [60]. Generally, microdialysis is used for small molecules like metabolites or small peptides, [61] while the proportion of high molecular weight proteins yield is very low (~1%) [62]. For this reason, microdialysis is better suited for metabolomic, rather than proteomic studies [63].

### 5.3. Wick Method

This method was adopted for the first time by Aukland and Fadness [64] to measure the osmotic pressure in tumor tissue. The method consists of placing a wick-in-needle acutely, with a pressure transducer [65,66]. The wick method, however, has several disadvantages such as bleeding, inflammatory reaction and cellular damage.

### 5.4. Glass Capillary

Sylven and Bois [67] used this method for the first time to obtain an IF sample. They inserted a capillary tube in the tumor periphery, rich in edema-like interstitial fluid. The method is relatively simple, but the liquid collected may not be true interstitial fluid.

### 5.5. Capillary Ultrafiltration

Ultrafiltration is generally used for the isolation of tissue fluids after implantation of capillary probes in vivo [68]. For the first time, this method was used for the isolation of the TIF from fibrosarcomas in mice [69] by using membranes with an molecular weight cut-off of 400 kDa. This method, however, has several limitations due to very low protein concentrations, [46] and does not represent the TIF composition [8].

## 6. Methods for Obtaining TIF from Fresh Tissue Specimens

### 6.1. Tissue Elution

Tissue elution is the most used method for TIF isolation. This method was developed by Celis et al. for the identification of biomarkers in breast cancer [54,70]. In this method, fresh biopsies isolated from women with breast cancer are cut in small pieces (1–3 mm), washed with a phosphatase saline buffer and incubated at 37 °C in a CO_2_ incubator. The supernatant collected after a 1h elution is the TIF. There are two major problems with this method: (1) The presence of major plasma proteins and (2) the contamination of the TIF by intracellular proteins. The presence of intracellular proteins is not a problem for biomarker identification, but may be a challenge for the identification of proteins actually present in the TIF. In gynecology, this method was used in ovarian cancer [71,72,73] and leiomyoma [74,75] for the exploration of the TIF by proteomics.

### 6.2. Tissue Centrifugation

Tissue centrifugation is another method [76] used for the isolation of the IF and the secretome. Originally, this method was used for the isolation of the TIF from tissues like the cornea [77] and the tail tendon, [78] which are rich in collagen. The method consists of exposing the tumor to an increased G-force to obtain an undiluted IF. The main question raised by the researchers was whether during centrifugation, if the liquid obtained was representative of the TIF. Using extracellular tracer ^51^Cr-EDTA, they concluded that it was. This method is suitable for the study of the TIF proteomics in ovarian cancer [79,80], and for the identification of biomarkers in endometrial cancer [80]. In addition to ovarian and endometrial cancers, this method has also been used for other cancers, for the identification of diagnostic and prognostic biomarkers.

## 7. TIF: A Biomarker Source in Gynecologic Cancer

Recently, with the advent of mass spectrometry (MS), many efforts have been made to identify biomarkers, especially in cancer tissues and in serum plasma [81,82,83,84]. To date, however, no biomarker from proteomic studies has been used in clinical practice. Serum/plasma is the main fluid used in biomarker research. The presence of abundant proteins such as albumin, IgG and transferrin makes it inadequate for the research of biomarkers [85,86]. Wang et al. were the first to use two dimensional gel electrophoresis (2-DE) and MS on TIF ovarian cancer. They identified eight possible biomarkers. Out of these, STIP1, LAP3, TPI1, UCHL1, BNDF and transferrin were validated by western blotting, immunohistochemistry (IHC) and enzyme-linked immunosorbent assay (ELISA). STIP1 was validated by ELISA in the serum of patients and was added to the list of possible diagnostic markers. Another study using 2-DE and MS was conducted by Cortesi et al. [72] identifying 58 proteins. Of them, only S100-A8 was validated, which is involved in cell cycle progression and differentiation [87]. Two other studies have been conducted using LC-MS/MS (liquid chromatography coupled with tandem mass spectrometry), in which 1338 proteins were identified in total and only PRDX1 was validated [71]. Haslene-Hox et al. [80] conducted a proteomic study on ovarian cancer TIF and found that WD-repeat containing protein 1 was overexpressed in the malignant ovarian tissue if compared to the healthy tissue, and suggested it could be a therapeutic target in ovarian carcinomas. As seen in data collected (Table 1), there are discrepancies in the results of the studies mentioned above, due to the different methods adopted for collecting the TIF, tumor grade, and the proteomic method used for protein quantification and identification [88]. To date, CA-125 is the only biomarker used in ovarian cancer. Its presence is very high in cancer tissue, while once released in the blood, the protein is diluted. The TIF can thus be considered as a treasure trove of potential biomarkers and the best choice for biomarker research [81,89]. Until now several studies were conducted for the identification of markers in gynecologic cancer. However, none of these markers has reached the validation phase. In our opinion, clinical studies with large number of patients are needed to validate these proteins.

We examined the publications listed in Table 1 to perform a pathway analysis of the proteins identified in these studies. By using the PANTHER Pathway Protein Classification system, we discovered that proteins in the IF of ovarian cancer belong to 15 biological pathways including: The adenine and hypoxanthine salvage pathway, axon guidance mediated by semaphorins, blood coagulation, cytoskeletal regulation by Rho GTPase, de novo purine biosynthesis, de novo pyrimidine ribonucleotide biosynthesis, glycolysis, Huntington’s disease, the integrin signaling pathway, Parkinson’s disease, the pentose phosphate pathway, the plasminogen activating cascade, purine metabolism, pyrimidine metabolism and the ubiquitin-proteasome pathway. We conducted the same analysis for the endometrial cancer IF. We discovered that proteins belong to five biological pathways, including: Cytoskeletal regulation by Rho GTPase, glycolysis, Parkinson’s disease, the pentose phosphate pathway, and the ubiquitin-proteasome pathway. This analysis shows the pathways in which the proteins identified in the TIF are involved. This information could be relevant not only for the study of biomarkers, but also for the study of mechanisms related to these diseases.

## 8. Tumor Interstitial Fluid in the Treatment of Cancer and Its Biological Implications

As we have already stressed above, the TIF plays an important role in cancer development. However, the mechanism through which the TIF influences tumor development has not been clearly ascertained to date. Several studies suggest that the tumor has a high uniform pressure that leads to neutralization of the normal difference in pressure between vessels and healthy tissue [90,91,92]. This causes an accumulation of drugs in the periphery of the tumor, leading to the failing of therapies [93]. The high P_if_ leads to a limited spread not only of drugs (low molecules < 1 kDa) but also of larger molecules such as monoclonal antibodies and nanoparticles, with the consequence being that these molecules rarely penetrate the area of the tumor [90,91,92,93,94]. Different strategies have been developed to alter the P_if_. The main strategy is based on anti-angiogenetic drugs which alter the tumor vasculature and decrease interstitial pressure after therapy, which blocks the formation of new blood vessels [95]. Hyperthermia is another strategy used to alter the P_if_ and consists of increasing the temperature of the tumor to reduce interstitial pressure. This strategy had a discrete success in melanoma [96] and multiple glioma [97]. However, the developed therapies are still experimental and have little benefits. The only real benefit in reducing the P_if_ was obtained from the use of vasoconstrictors like angiotensin H receptor antagonists with conventional therapies [98]. The use of vasoconstrictors leads to a reduction in blood flow, and a consequent reduction in the P_if_ and an alteration in the distribution of the blood flow inside the tumor, finally leading to tumor hunger [94]. Another strategy in cancer treatment is the manipulation of the flow of the IF, with a consequence of blocking tumor growth. The most important studies are related to chemotaxis, as an event involving several molecules (chemokine/chemokine receptors) taking part in the modulation of flow-enhanced invasion [99,100]. Bevacizumab, as an anti-neoplastic therapy, could act by interfering with this mechanism and subsequently improve oncological outcomes [101]. Inhibition of integrins and degradation of other glycans leads to a reduction of cancer metastatization [102], and these molecules could be targets for future cancer treatments. To date, proteomic studies of the TIF have identified several potential clinical biomarkers. Proteomic approaches could be used to quantify known bioactive compounds in the TIF [103,104,105,106], in which numerous posttranslational modifications occur. Garvin S and Dabrosin C [107] used microdialysis to sample the TIF from solid breast tumors in situ, where VEGF is biologically active. For the first time, they showed that tamoxifen decreased extracellular VEGF in vivo in solid MCF-7 tumors in nude mice. Wiig et al. isolated bone marrow interstitial fluid (BMIF) in rats and humans by centrifugation [108]. They isolated BMIF from bone marrow biopsies obtained at diagnosis and at two to four weeks after the start of the induction therapy. They found that acute myeloid leukemia (AML)-derived BMIF, but not plasma, repressed hematopoietic progenitor cell growth. This effect was lost after successful induction treatment [109]. The experiments of Iversen PO and Wiig H [109] suggest a mechanistic role of TNF α (Tumor Necrosis Factor α) and adiponectin in AML. The levels of this cytokine diminished in BMIF in patients in remission, while plasma levels were unaffected by the therapy. The research by Iversen PO and Wiig H is a clear example, showing that quantification of substances in the IF may give important information on disease progression. Two other studies [110,111] have shown the importance of targeting syndecan-1 heparan sulfate proteoglycan in the microenvironment, and not in plasma multiple myeloma.

## 9. Conclusions

To date, no biomarker identified through proteomic analyses is yet ready to be used in clinical practice. However, the TIF represents a potential source of biomarkers, and its molecular analysis will likely increase our knowledge about tumoral pathogenesis. In this review, we have analyzed recent studies on the tumor interstitium and the composition and formation of the TIF. Only a few studies dealing with the proteome of the IF of gynecologic cancers have emerged in recent years, showing a particular interest in ovarian cancers. These studies have been analysed in this review. There are few validated biomarker candidates in the presented studies. Until now, no standard method has been identified for TIF isolation. Thus, the method chosen for fluid isolation may have a direct impact on the results of the proteomic studies conducted. For the identification of the markers, the proteomic data of the TIF should be integrated into a wider platform also involving cancer cell lines, tumor tissue, animal models, genomic and transcriptomics [100]. Developing such a strategy could open new frontiers and opportunities in the discovery of cancer biomarkers.

## Figures and Tables

**Table 1:** Summary of proteomic studies in gynecologic cancer, including clinical biomarker candidates chosen for validation.

Cancer Type	Isolation Technique	Sample	Candidates	Validation Methods	Published Protein Findings	Reference
Ovarian carcinoma	Tissue elution	TIF, NIF	*UCHL1*, *STIP1*, *LAP3*, *TPI1*	WB, IHC	8 proteins identified by 2-DE and MS	[73]
			Transferrin, *BNDF*	ELISA		
		Serum (for data validation)	*STIP1*	ELISA		
Ovarian carcinoma	Tissue elution	TIF, NIF	*S100-A8*	IHC	58 proteins identified by 2-DE and MS	[72]
Ovarian carcinoma	Centrifugation	TIF, Plasma (patient; control)	No protein validation		769 proteins identified by LC-MS/MS. 124 in patient plasma and 102 in control plasma	[79]
Ovarian carcinoma	Tissue elution	TIF/ascites	*PRDX1*	WB	569 proteins identified	[71]
		Serum (normal/benign)	*PRDX1*	ELISA		
Ovarian carcinoma	Centrifugation	TIF	*WDR1*	WB, SRM	650 proteins identified	[80]
Endometrial cancer	Centrifugation	TIF	No protein validation		399 proteins identified	[80]

Abbreviations: TIF—tumor interstitial fluid; NIF—normal interstitial fluid; SRM—single reaction monitoring; IHC—Immunohistochemistry; LC-MS/MS—liquid chromatography coupled with tandem mass spectrometry; WB—western blotting.
